# An Overview of Sex Bias in *C. neoformans* Infections

**DOI:** 10.3390/jof4020049

**Published:** 2018-04-18

**Authors:** Tiffany E. Guess, Joseph A. Rosen, Erin E. McClelland

**Affiliations:** Department of Biology, Middle Tennessee State University, Murfreesboro, TN 37132, USA; tiffanyeguess@gmail.com (T.E.G.); jar8n@mtmail.mtsu.edu (J.A.R.)

**Keywords:** *Cryptococcus*, *neoformans*, immune privilege, sex susceptibility, sex hormones, sexual dimorphism, testosterone, estrogen

## Abstract

Cryptococcosis, a fungal disease arising from the etiologic agent *Cryptococcus neoformans*, sickens a quarter of a million people annually, resulting in over 180,000 deaths. Interestingly, males are affected by cryptococcosis more frequently than females, a phenomenon observed for more than a half century. This disparity is seen in both HIV^−^ (~3M:1F) and HIV^+^ (~8M:2F) populations of cryptococcal patients. In humans, male sex is considered a pre-disposing risk factor for cryptococcosis and males suffering from the disease have more severe symptoms and poorer outcomes. There are numerous observational, clinical and epidemiological studies documenting the male disadvantage in *C. neoformans* but with no further explanation of cause or mechanism. Despite being commonly acknowledged, little primary research has been conducted elucidating the reasons for these differences. The research that has been conducted, however, suggests sex hormones are a likely cause. Given that the sex difference is both prevalent and accepted by many researchers in the field, it is surprising that more is not known. This review highlights the data regarding differences in sexual dimorphism in *C. neoformans* infections and suggests future directions to close the research gap in this area.

## 1. Introduction

*Cryptococcus neoformans* is an encapsulated yeast that causes fungal meningitis in immune compromised persons. While it is primarily described as an AIDS-defining illness [1], it causes disease in other immune compromised populations such as those undergoing organ transplantation, chemotherapy, or other immunosuppressive regimens [2]. One of the interesting epidemiological aspects of this yeast is the increased incidence of the disease in males (2–3:1 males:females), which was noted before the start of the HIV epidemic [3]. One of the first studies to describe this biological sex difference was a case report and review of the literature. In 1966, Campbell reported that 82% of patients with pulmonary cryptococcosis were male [4]. Another case study published in 1970 described 29 patients with cryptococcal CNS disease in which 68% of patients were male [5]. Before 1990, a number of other case studies documented the same finding [6,7] but the onset of the AIDS epidemic drastically highlighted the sex difference, such that in 1995, Manfredi et al. published a paper asking “Is AIDS-related cryptococcosis more frequent among men?” [8]. Since then, numerous papers and case studies from around the world have documented that males are at increased risk for infection with *Cryptococcus* and ≥70% of patients with cryptococcosis are male, depending on the region. A recent retrospective study in the US found that not only was there increased incidence of *Cryptococcus* in males but males were three times more likely to be hospitalized and four times more likely to be hospitalized if they had AIDS. Males were also three times more likely to die [9]. Most recently, a 20-year longitudinal study out of Colombia reported greater frequencies of *C. neoformans* infection in both HIV^+^ (5.4M:1F) and HIV^-^ (3.9M:1F) [10] Cryptococcosis presents either in its most lethal form as cryptococcal meningitis or as a non-meningeal infection: pulmonary, cutaneous, or cryptococcaemia (a *C. neoformans* blood infection) [11]. With the exception of one study indicating that female gender was associated with higher mortality in cryptococcaemia [12], when a sex bias is observed in cryptococcal studies, the prevalence is consistently more common in males, no matter its presentation [13,14,15,16,17,18,19,20,21,22,23,24,25,26,27,28,29].

What might explain this? Both prior to the HIV epidemic and recently, a common explanation for the increased incidence of disease in males was increased exposure to *C. neoformans* [13,30,31]. Since *C. neoformans* has been isolated from pigeon droppings, trees and the soil and men are more likely to work outside of the home, this could explain some part of the sex susceptibility difference [32]. However, studies have found that males and females are infected at equal rates [31,33,34]. Noting higher infection rates in men, researchers in the 1970s conducted skin reactivity tests from samples obtained in Oklahoma, which showed equal numbers of exposed males and females [34]. More recent serology testing from The Bronx, NY indicated exposure to *C. neoformans* is extremely common in both male and female children alike [33]. In addition, serology data from the healthy donors used in McClelland et al. (2013) found that more females than males were positive (1.4F:1M, unpublished data), suggesting that environmental exposure is not the only explanation [35]. Another hypothesis proffered for the increased disease in males is patient non-compliance with antifungals [36,37]. While patient non-compliance may account for some part of increased cryptococcosis in men, since there doesn’t seem to be any quantifiable data on this, it is difficult to say how much it contributes.

Another possible explanation for increased incidence of disease in males is that the observed sex bias simply reflects HIV status, as males tend to have more risk-taking behavior, which increases their probability of contracting HIV. It would then follow that since more males have HIV, greater numbers will progress to AIDS and get cryptococcosis. While it is true that 76% of people living with HIV currently are male [38], it has also been shown that females are both more susceptible and have increased mortality from HIV compared to males (see a review on this topic in [39]). Additionally, it has been reported that sexually transmitted diseases occur more frequently and severely in women during their reproductive years supposedly due to behavior, sex-related mechanisms in reproduction and sex-specific steroid hormone levels [40]. Since it is hypothesized that testosterone is linked to increased risk-taking behavior, one could also explain the increased numbers of men with HIV with the fact that testosterone is a known immune-suppressant [41,42,43]. There are a number of studies that show an increased immune response in castrated male mice compared to intact male and female mice, suggesting that testosterone can suppress the immune response [44,45]. This could also explain why male sex is a risk factor for immunocompetent patients with cryptococcosis [46] and immunocompetent males are more likely to get primary cutaneous cryptococcal infections (17:4, M:F) [47].

In contrast, estrogen is known to enhance the immune response [48,49]. During *C. neoformans* infections, specifically, phagocytosis by host cells rose in the presence of diethylstilbestrol, a synthetic estrogen compound in one study [50]. Another experiment showed human female macrophages phagocytosed greater numbers of *C. neoformans* than did their male counterparts [35]. Thus, there may be a decreased incidence of females with cryptococcosis, at least in part, because they have higher levels of estrogen. Some anecdotal evidence to support this is that female patients with cryptococcal meningitis are more likely to have other comorbidities that depress the immune response, such as systemic lupus erythematosus [51], which is often associated with T-cell abnormalities [52]. In addition, Tamoxifen, a selective estrogen receptor modulator (SERM), has shown anti-cryptococcal properties [53], not because it modulates estrogen binding but because it interferes with *C. neoformans* calmodulin signaling. Tamoxifen is fungicidal in vitro and is currently being studied for its efficacy as a treatment for cryptococcal meningitis in combination with amphotericin B (AmpB) and fluconazole [54].

So, while increased environmental exposure is often touted as the explanation for the observed gender bias in *C. neoformans* infections, it is entirely possible that there is a biologic effect, as noted by Micol et al. in 2007. That study looked at Cambodian HIV patients and found that the increased disease observed in males was “independent of occupation, residence and degree of HIV-related immunosuppression, suggesting a sex-related biologic effect” [28].

## 2. Primary Research

Despite the clear sex susceptibility difference in case studies and patient populations, there are only a few publications with actual data to help explain why females fare better during *Cryptococcus* infections. A trio of papers published in the 1970s are considered the basis for sex bias studies in *C. neoformans.* Prior to the HIV crisis, researchers noted the increased frequency of cryptococcosis in males despite evidence indicating males and females were exposed at similar rates [34]. In an attempt to determine the cause of this gap, Mohr et al. tested the effects of hormones on seven human *C. neoformans* isolates [31]. After six days of incubation, growth was completely inhibited in all isolates incubated with diethylstillbesterol (10 µg/mL) [31]. When incubated with estrogen (1 μg/mL), three isolates exhibited complete inhibition and the remaining four showed markedly inhibited growth [31]. There was no growth inhibition observed in samples incubated with progesterone, testosterone, or dorethynodrel, a synthetic progestin [31]. The concentration of estrogen used in this study was higher than the concentration occurring in human females. The authors note that inhibitory effects of estrogens are likely not solely responsible for the lower levels of cryptococcosis in females but may partially explain the differences in incidence between the sexes [31]. 

In a follow-up study using the same isolates, Mohr tested inhibition of *C. neoformans* when incubated with AmpB and hormones [55]. Results showed complete inhibition of all isolates incubated with diethylstillbesterol (0.5 µg/mL + 0.3 µg/mL AmpB) [55]. Estradiol (0.01 µg/mL plus 0.3 µg/mL AmpB) completely suppressed the growth of four isolates [55]. Either compound alone failed to suppress the growth completely [55]. Progesterone plus AmpB showed slight inhibition and there were no inhibitory effects with testosterone [55]. The effective concentration of estradiol was much lower than that of diethylstilbesterol or AmpB alone, providing direct evidence of the inhibitory effects of estrogen on *C. neoformans*. 

To further discern the efficacy of estrogens in conjunction with AmpB, the same researchers in the aforementioned studies collected blood samples from 11 cryptococcal meningitis patients before, during and after treatment of AmpB and before, during and after administration of 5 mg diethylstilbesterol [50]. The samples were infected with a non-encapsulated strain of *C. neoformans* and phagocytosis recorded. Phagocytic activity of all patient samples were markedly depressed both before and during treatment of AmpB [50]. In patients receiving diethylstilbesterol, however, phagocytic activity increased significantly [50]. Further, antigen titers decreased in all patients administered the synthetic estrogen [50]. When administration of the estrogen was stopped in one patient, antigen titers increased and there was an abrupt decline in the percent phagocytosis of the patient’s blood samples [50]. The results of these studies indicate that estrogens play a dual role in *C. neoformans* infections by both inhibiting growth of the pathogen as well as increasing phagocytic activity of host immune cells. 

After the Mohr’s work in the 1970’s, we can find no other research papers looking into the male/female inequality in *C. neoformans* infections for nearly three decades despite the disparity growing larger during the AIDs epidemic of the 1980s and 1990s. The next paper was published in 2002 and suggested that female outbred mice fared better than males because they had higher levels of the cytokines TNF-α and IFN-γ in the blood and spleen during *C. neoformans* infection, suggesting a more protective Th1 immune response in females [56]. 

Another paper asked the question of whether sex contributed to a *C. neoformans* infection in the *C. elegans* invertebrate model [57]. While *C. elegans* is primarily hermaphroditic, males are occasionally produced and, interestingly, are more resistant to a *C. neoformans* infection than hermaphrodites [58]. This resistance was found to be correlated with increased activity of the DAF-16 stress-response transcription factor (also associated with increased longevity in *C. elegans*), suggesting that resistance to *C. neoformans*, at least in *C. elegans*, is transcriptionally regulated [58].

In 2007, a five-year observational study from France reported that male gender, along with positive HIV status and infection of serotype A (rather than serotype D) *C. neoformans* is a major determinant of presentation and outcome of cryptococcosis [59]. Enrolled patients were either HIV^+^ or HIV^-^ that had at least one positive *C. neoformans* culture from urine, blood, or cerebral spinal fluid (CSF). Of the 230 patients enrolled in the study, 78% were male and 62% were both male and HIV^+^ [59]. Of the HIV^+^ population, males had a greater incidence of fungaemia, positive urine cultures and more disseminated infections [59]. CD4^+^ T cells were approximately the same in males and females. In the HIV-population, the only difference seen between sexes was higher CSF antigen titers in males [59]. Based on the patients in this study, cryptococcosis was more severe in men than women and those differences were more pronounced in the HIV^+^ population.

In a group of experiments published in 2013, researchers examined both host and pathogen features as they pertain to sex. Using a cryptococcal meningitis^+^/HIV^+^ patient cohort from Botswana [60], they found that males had higher mortality rates than females despite having increased numbers of CD4^+^ T cells at the time of hospitalization [35]. Corresponding *C. neoformans* strains isolated from the CSF of those patients showed strains isolated from females released more capsular glucuronoxylomannan (GXM) and had longer doubling times than strains isolated from males [35]. When incubated with exogenous testosterone, however, strains showed increased GXM release suggesting that exposure to a male environment may increase the virulence of a *C. neoformans* infection [35]. Looking at the innate immune response in healthy donors to *C. neoformans* infection, they found macrophages isolated from females phagocytosed higher numbers of *C. neoformans* than macrophages isolated from males, yet male macrophages had a higher fungal burden and were killed at increased rates by *C. neoformans* compared to female macrophages [35]. Additionally, after a chronic cryptococcal infection, male Balb/c mice had a significantly higher splenic fungal burden than did female mice [35]. This data suggests that the interaction between *C. neoformans* and the immune response within different host sex environments contributes to the increased prevalence of cryptococcal meningitis in males. See Table 1. 

## 3. Observational Studies

Given the data above, we wondered if other animals displayed the sexual dimorphic susceptibility observed in mice and humans. There were no differences in infections found in koalas [61], dogs [62,63,64] and dolphins [65]. There may be a sex difference in infections in birds but since not all the birds could be sexed, this is still unknown [66]. The picture in cats appears to be more complex. A large retrospective study conducted at the University of Sydney found that male and female cats (N = 144) were infected approximately equally (53%:47%, M:F) [63]. However, studies done at the University of Pennsylvania [67] (N = 19), the University of Georgia [68] (N = 35) and a smaller case study done at the University of Sydney [69] (N = 27) found that male cats were affected more often than female cats. One possibility for the difference may be the smaller sample sizes for the University of Pennsylvania, the University of Georgia and the first University of Sydney papers. Another possibility is that there is a sex difference only when cats are infected with *C. neoformans* versus *C. gattii*. The studies conducted in the USA involved infections with *C. neoformans*, while the studies from Australia involved infections from both *C. neoformans* and *C. gattii*. In the first study from the University of Sydney [69] (N = 27), *C. neoformans* was isolated from 21 cats, while *C. gattii* was identified in the remaining six (~22%). When the authors went back and did the retrospective study with the larger sample size, about 30% of cats were infected with *C. gattii*. Since the data was not broken down by sex and serotype, it is possible that there were more male cats than female cats infected with *C. neoformans*. This should be explored further.

## 4. Other Fungi Exhibiting Sexual Dimorphism in Infection 

There are other fungi that exhibit sexual dimorphism in infection. Similar to *C. neoformans*, *Paracoccidioides brasilienis,* the etiologic agent of paracoccidioidmycosis (PCM), exhibits gender susceptibility during infection with males more likely to suffer overt disease than females (11–30M:1F) [70,71,72]. Also echoing *C. neoformans, P. brasilienis* is found frequently in soil and most often afflicts agricultural workers. Researchers initially hypothesized that males suffered disease in greater numbers due to increased exposure. However, that is not the case, with skin test results showing equal rates of infection [73]. Although the mechanism of action is still unknown, multiple studies point to sex hormones playing a key role in the differences of PCM seen in males and females, particularly estrogen levels. In one cohort, 70% of the women diagnosed with PCM were menopausal, which is characterized by many symptoms including decreased estrogen production [74]. Further, the sex bias does not exist in children suffering from PCM, with males and females suffering at similar rates. The sex differences are only observed in patients around the age of puberty, 13, upward [75,76]. Microarray analysis revealed incubation of 17β-estradiol with *P. brasilienis* results in the up or down regulation of over 500 genes [77] and results of binding studies are suggestive of a hormone-binding protein in the cytosol of this yeast [78]. Increased levels of estrogen clearly appear to confer protection among people exposed to *P. brasilienis* but more research needs to be done to understand the mechanism as well as host-pathogen interactions as they relate to sex hormones.

Unlike *C. neoformans* and *P. brasilienis*, *Candida albicans* occurs with greater frequency and severity in females (1M:3–5F) [79,80,81,82]. Considered typical gut and mouth flora, *C. albicans* can act as an opportunistic pathogen and overgrow to the point of infection. It is the main cause of vaginal candidiasis but can also infect the mouth, throat and bloodstream [2,83,84]. The reversal of the sex bias in *C. albicans*, compared to *C. neoformans* and *P. brasilienis* may be explained in a few ways. First, the female anatomy puts women at a greater risk for genital candidiasis [85]. Second, while estrogens are known immunostimulators, there is a noted exception—the female reproductive tract. Research shows estrogen decreases the expression of several cytokines and NF-κB in the uterine and vaginal epithelium, suggesting that the hormone may be a key factor in weakening female host defenses in the face of opportunistic microflora such as *C. albicans* [86,87]. Increased levels of estrogen during pregnancy, the use of oral contraceptives and hormone replacement therapy have all been positively associated with increased *C. albicans* infection [88,89]. However, more is understood about the mechanisms of action in this yeast than the previous two discussed. An estrogen binding protein (Ebp1) located in the cytosol of *C. albicans* binds host estrogen, specifically 17β-estradiol with a high affinity [90]. Further, one study found that *C. albicans* cells treated with estrogen survive at higher temperatures (48 °C) by upregulating a heat-stress protein (Hsp90) better than cells not treated with estrogen [91]. The same study demonstrated increased levels of *Candida* multidrug resistance (*CDR1*) mRNA in the presence of estrogen rather than control cells [91]. Similar to *C. neoformans* and *P. brasilienis*, *C. albicans* is influenced by the host hormonal environment. Fortunately, we have a greater understanding of how hormones affect this pathogen. This knowledge could help researchers trying to uncover mechanisms in other fungi with sex biases during infection.

## 5. Conclusions

A phenomenon accepted in the *C. neoformans* community, yet rarely the focus of study, the discrepancy between males and females suffering from cryptococcosis has been well documented since the 1960’s. However, due to the various possible causes (increased exposure in males, increased numbers of male HIV patients and patient non-compliance), some researchers in the community do not believe there could be a biologic reason for the increased incidence of disease in men. Men suffer disease and death from *C. neoformans* at higher rates than women, however, the reasons why remain ambiguous. Of the thousands of peer-reviewed articles on this pathogen, only seven provide data to help explain potential causes of this sexual dimorphism in infection. The research suggests that explanations are likely biologic and not just differences in exposure rates, numbers of male HIV patients or medical non-compliance. Further, the study published in the 1970s and 2013 point to sex hormones playing a role in both the immune response of the host to a *C. neoformans* infection and the effect of the hormonal environment on *C. neoformans* virulence [35]. Sex hormones have been implicated in sex biases to infection in a number of pathogens, a few of which were discussed above. *C. neoformans* may follow suit, although we are a long way from having conclusive evidence and even further from understanding the mechanisms of action. Future research should investigate the effects of sex hormones on the interaction of *C. neoformans* with males or females to determine how/if they affect pathogenesis. If so, the molecular mechanisms for those effects need to be elucidated. Much work is still needed to unravel the complexities of the male/female host-pathogen relationship in cryptococcosis. 

## Figures and Tables

**Table 1:** A summary of major findings from the primary research papers reviewed above.

Published	Author	Organism Studied	Major Findings
1972	Mohr et al.	*C. neoformans*	Growth of clinical isolates was inhibited when incubated with either a synthetic or natural human estrogen.
1973	Mohr et al.	*C. neoformans*	Estrogens, when combined with AmpB, markedly inhibited *C. neoformans* growth in vitro.
1974	Mohr et al.	Humans	Phagocytic activity increased and antigen titers decreased in cryptococcal meningitis patients administered synthetic estrogen.
2002	Lortholary et al.	Mice	Females had increased levels of the helpful Th1 cytokines TNF-α and IFN-γ in blood and spleen during *C. neoformans* infection.
2006	van den Berg et al.	*C. elegans*	Males were found to be more resistant to *C. neoformans*. This resistance was linked to increased activity of the DAF-16 stress-response transcription factor.
2007	Dromer et al.	Humans	Male gender was a major determinant of outcome during *C. neoformans* infection. Cryptococcosis was more severe in men.
2013	McClelland et al.	Mice, Humans	Spleens of male mice showed higher fungal burden than female mice after chronic cryptococcosis infection. Human males had higher CD4^+^ T cells yet had higher mortality rates. Macrophages isolated from females were more effective during a *C. neoformans* infection than male macrophages.
